# Reconsidering neuraxial analgesia at end of life: Clinical, ethical, and socioeconomic perspectives^☆^

**DOI:** 10.1016/j.inpm.2025.100610

**Published:** 2025-07-02

**Authors:** Sanjeet Narang, Jason Yong, David Hao

**Affiliations:** a Department of Anesthesia, Critical Care, and Pain Medicine, Beth Israel Deaconess Medical Center, Harvard Medical School, Boston, MA, USA; b Department of Anesthesiology, Critical Care, and Pain Medicine, Brigham and Women's Hospital, Harvard Medical School, Boston, MA, USA; c Department of Anesthesia, Critical Care and Pain Medicine, Massachusetts General Hospital, Harvard Medical School, Boston, MA, USA

**Keywords:** Neuraxial, End-of-life, Cancer pain, Epidural, Intrathecal, Palliative

## Abstract

Pain is one of the most prevalent and distressing symptoms experienced by patients nearing end of life, particularly among those with cancer. While systemic opioids are the mainstay of treatment, their limitations necessitate consideration of alternative strategies. Neuraxial analgesia, including epidural and intrathecal drug delivery systems, offers targeted pain relief with reduced systemic burden. Yet despite supportive data, these interventions remain underutilized due to clinical, ethical, logistical, and socioeconomic barriers. This article examines the complex decision-making involved in offering neuraxial analgesia at the end of life, weighing risks and benefits, shifting patient goals, and the challenges of care coordination. By reframing neuraxial analgesia not as an extraordinary measure, but as a legitimate and potentially transformative option, we advocate for broader, more equitable integration of these therapies.

## 1 Introduction

Pain remains one of the most feared and burdensome symptoms for patients nearing end of life, with data showing that 81 % of patients with cancer experience pain in their last week of life. Among these patients, nearly one-third experience severe pain. While oral and intravenous opioids remain the cornerstone of pain relief, this approach often leads to significant side effects and, despite escalating doses, still leaves some patients in distress.

Neuraxial analgesia, including epidural and intrathecal therapies, can offer targeted relief with less systemic toxicity. Meta-analyses show that intrathecal drug delivery systems (IDDS) can reduce pain intensity [1]. Similarly with epidural analgesia, some studies suggest meaningful reduction in opioid requirements and improved pain control, though data is limited and heterogeneous [2]. In pediatric populations, Anghelescu et al. highlight how continuous catheter-based techniques can ease suffering and support death in the patients preferred setting [3]. Despite their potential benefits, these therapies remain underutilized, primarily because of technical complexity, the challenges of managing end-of-life care, and numerous systemic and logistical hurdles that hinder implementation.

We aim to reflect on these challenges, highlight key considerations, and offer a balanced perspective to support thoughtful, compassionate decision-making in this delicate, yet vital, aspect of care. By bringing these often-sidelined conversations to the forefront, we hope to foster greater awareness, dialogue, and collaboration, ensuring that the full potential of these therapies is considered in service of patients and their goals of care.

## 2 Clinical and ethical considerations

Choosing neuraxial analgesia at the end of life requires more than a technical assessment of pathology. It demands a careful, patient-centered approach that equally considers individual goals, values, and definitions of comfort. A persistent tension in end-of-life care is the balance between the desire to alleviate suffering and the fear of subjecting a patient to another invasive intervention. This dichotomy is not easily reconciled.

Presenting patients with all available options, along with their respective risks and benefits, and likely complications and outcomes, helps set realistic expectations and empowers them to make informed, value-aligned decisions. When considering neuraxial interventions, we carefully weigh the balance between IDDS and epidural infusions: IDDS offers steady, long-lasting relief but requires more time for adjustment and closer clinical oversight, while epidurals are generally quicker to initiate and maintain but may tether patients to cumbersome external equipment that can be distressing in their final days. Recognizing that each option involves trade-offs tailored to the individual, we must remain ready to reassess decisions as the patient's condition evolves.

These decisions are further shaped by uncertainty inherent in prognostication. Although epidural analgesia is generally reserved for patients with very limited life expectancy, survival predictions in advanced cancer are often imprecise, and clinicians may mis-estimate survival, particularly in the final weeks and months. This ambiguity can result in approaches that are either overly aggressive and burdensome, or overly cautious and result in withholding meaningful pain relief [4]. Patients in these contexts frequently have multiple comorbidities that increase the risk of procedural complications, and at times, traditional contraindications such as infection, coagulopathy or spinal metastases are outweighed by the imperative to relieve suffering [3]. Continuous reassessment remains essential, as the decision to proceed demands a careful balance between the intervention's potential risks and the patient's urgent need for pain control. These risks carry serious potential sequelae. For example, performing an epidural injection on a patient with an elevated INR might result in an epidural hematoma, requiring urgent neurosurgical decompression or hastening the end.

This is the art of medicine, an inherently nuanced conversation that asks us, as clinicians, to hold these complex, sometimes conflicting considerations in balance. We must distill these layers into actionable choices for patients and families. We must acknowledge the technical challenges, the need for careful coordination across care teams, and the urgency of responding to rapidly changing circumstances. Too often, the prospect of invasiveness, logistical burdens, and technical complexity overshadows the potential for profound pain relief and a more dignified death, so we must bring these reflections to the surface through transparent, thoughtful discussions that honor both clinical realities and patients’ deeply personal narratives.

## 3 Socioeconomic considerations

Beyond clinical decision-making, access to neuraxial analgesia is heavily influenced by socioeconomic factors and systemic barriers. For individuals with a life expectancy beyond three to six months, intrathecal therapy has been associated with reduced healthcare utilization and costs [5]. However, for those with shorter prognoses, external tunneled peripheral or epidural catheters may offer a more pragmatic and less burdensome solution, prioritizing effective relief without the expense or technical demands of fully implanted systems and potentially allowing patients to spend more meaningful time at home with family.

Yet these choices do not occur in a vacuum. Reimbursement challenges and administrative complexity often delay or prevent timely access. Insurance approvals can be inconsistent and onerous, particularly for patients receiving care outside major academic institutions. Duarte et al. have documented that access to intrathecal pumps in England remains clustered in a handful of academic centers, leaving significant gaps for patients elsewhere [6]. Disparities in infrastructure, clinical expertise, and awareness, highlighted by Pérez et al. and Barnosky et al., further compound these access challenges, widening inequities in care [7,8].

These considerations underscore that the question of offering neuraxial analgesia is never purely clinical. It is shaped by broader socioeconomic forces and the realities of how healthcare systems are structured and resourced in a given geographical location. Balancing these dimensions requires clinical judgment and thoughtful financial stewardship, as well as a commitment to equity, ensuring that relief from suffering is not contingent on geography, institutional affiliation, or administrative luck.

## 4 Conclusion

Neuraxial analgesia is not merely a technical intervention, it is a testament to our collective commitment to ease suffering at the end of life. As we navigate the uncertainties and complexities of each patient's final journey, we must ensure that these conversations and considerations are not hidden in the margins but brought to the forefront of compassionate, evidence-informed care. In doing so, we can honor the patient's values and goals, bridging the gap between what is clinically possible and what is practically achievable, always with the singular aim of reducing suffering in the most humane and dignified way possible.

## Declaration of competing interest

The authors declare the following financial interests/personal relationships which may be considered as potential competing interests: Sanjeet Narang reports a relationship with AIS Healthcare that includes: consulting or advisory. Sanjeet Narang reports a relationship with Medtronic Inc that includes: consulting or advisory. R. Jason Yong reports a relationship with Medtronic Inc that includes: consulting or advisory. If there are other authors, they declare that they have no known competing financial interests or personal relationships that could have appeared to influence the work reported in this paper.
